# Correction: Native *Wolbachia* from *Aedes albopictus* Blocks Chikungunya Virus Infection *In Cellulo*

**DOI:** 10.1371/journal.pone.0134069

**Published:** 2015-07-29

**Authors:** Vincent Raquin, Claire Valiente Moro, Yoann Saucereau, Florence-Hélène Tran, Patrick Potier, Patrick Mavingui

## Notice of Republication

This article was republished on July 9, 2015, to replace an incorrectly published version. Please download this article again to view the correct version. The originally published, uncorrected article and the republished, corrected article are provided here for reference.

## Supporting Information

S1 File Originally published, uncorrected article. (PDF)

S2 File Republished corrected article. (PDF)
