# Supplementary figures and images for: Native Wolbachia from Aedes albopictus Blocks Chikungunya Virus Infection In Cellulo
Source: PLoS One. 2015 Apr 29;10(4):e0125066. doi: 10.1371/journal.pone.0125066 (PMC4414612; doi:10.1371/journal.pone.0125066)

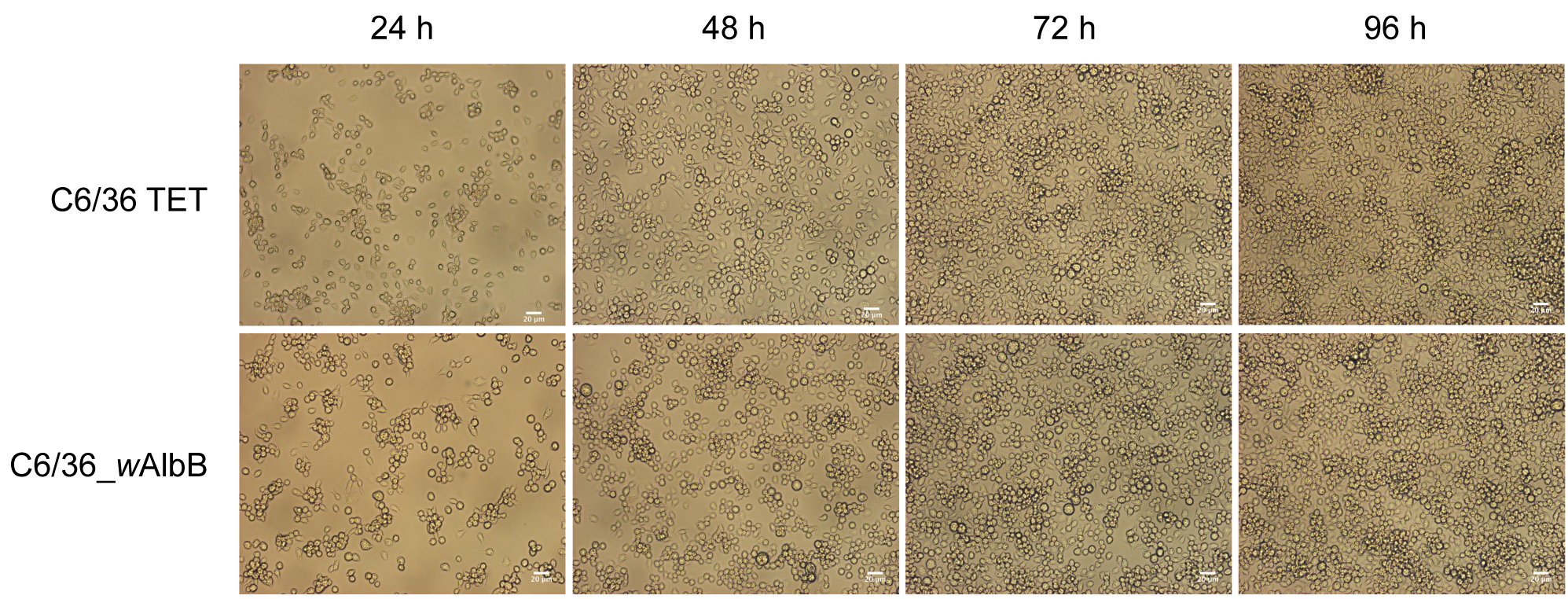

Supplement: S1 Fig — Pictures in light microscopy of C6/36 cells infected by Wolbachia (C6/36_wAlbB) or tetracycline-treated (C6/36_TET) during their growth in F25 cm2 flasks, between two passages (bars = 20 μm). (TIF) [file pone.0125066.s001.tif]
