# Supplementary material for: Correction: Native Wolbachia from Aedes albopictus Blocks Chikungunya Virus Infection In Cellulo
Source: PLoS One. 2015 Jul 29;10(7):e0134069. doi: 10.1371/journal.pone.0134069 (PMC4519297; doi:10.1371/journal.pone.0134069)
Supplement: S2 File — (PDF) [file pone.0134069.s002.PDF]

PLOS ONE

RESEARCH ARTICLE

Native *Wolbachia* from *Aedes albopictus* Blocks Chikungunya Virus Infection *In Cellulo*

Vincent Raquin[1¤]*, Claire Valiente Moro[1], Yoann Saucereau[1], Florence-Hélène Tran[1], Patrick Potier[1], Patrick Mavingui[1,2]*

1 Université de Lyon, UMR 5557 Ecologie Microbienne, CNRS, USC1190 INRA, VetAgro Sup, Université Lyon 1, Villeurbanne, France, **2** Université de La Réunion, UMR PIMIT, INSERM U1187, CNRS 9192, IRD 249, Plateforme de Recherche CYROI, Saint-Denis, La Réunion, France

¤ Current address: Insect-Virus Interactions group, Genomes & Genetics Department, Institut Pasteur, Paris, France
* vincent.raquin@gmail.com (VR); patrick.mavingui@univ-lyon1.fr (PM)

CrossMark
click for updates

Abstract

Wolbachia, a widespread endosymbiont of terrestrial arthropods, can protect its host against viral and parasitic infections, a phenotype called "pathogen blocking". However, in some cases *Wolbachia* may have no effect or even enhance pathogen infection, depending on the host-*Wolbachia*-pathogen combination. The tiger mosquito *Aedes albopictus* is naturally infected by two strains of *Wolbachia*, *w*AlbA and *w*AlbB, and is a competent vector for different arboviruses such as dengue virus (DENV) and Chikungunya virus (CHIKV). Interestingly, it was shown in some cases that *Ae*. *albopictus* native *Wolbachia* strains are able to inhibit DENV transmission by limiting viral replication in salivary glands, but no such impact was measured on CHIKV replication *in vivo*. To better understand the *Wolbachia*/CHIKV/*Ae*. *albopictus* interaction, we generated a cellular model using *Ae*. *albopictus* derived C6/36 cells that we infected with the *w*AlbB strain. Our results indicate that CHIKV infection is negatively impacted at both RNA replication and virus assembly/secretion steps in presence of *w*AlbB. Using FISH, we observed CHIKV and *w*AlbB in the same mosquito cells, indicating that the virus is still able to enter the cell in the presence of the bacterium. Further work is needed to decipher molecular pathways involved in *Wolbachia*-CHIKV interaction at the cellular level, but this cellular model can be a useful tool to study the mechanism behind virus blocking phenotype induced by *Wolbachia*. More broadly, this underlines that despite *Wolbachia* antiviral potential other complex interactions occur *in vivo* to determine mosquito vector competence in *Ae*. *albopictus*.

OPEN ACCESS

Citation: Raquin V, Valiente Moro C, Saucereau Y, Tran F-H, Potier P, Mavingui P (2015) Native *Wolbachia* from *Aedes albopictus* Blocks Chikungunya Virus Infection *In Cellulo*. PLoS ONE 10 (4): e0125066. doi:10.1371/journal.pone.0125066

Editor: Kostas Bourtzis, International Atomic Energy Agency, AUSTRIA

Received: January 4, 2015

Accepted: March 20, 2015

Published: April 29, 2015

Copyright: © 2015 Raquin et al. This is an open access article distributed under the terms of the Creative Commons Attribution License, which permits unrestricted use, distribution, and reproduction in any medium, provided the original author and source are credited.

Data Availability Statement: All relevant data are within the paper and its Supporting Information files.

Funding: This work was partially funded by the Agence Nationale de la Recherche (ANR, http://www.agence-nationale-recherche.fr/) grant ANR-2010-BLAN-170101 (ImmunSymbArt), the Fondation pour la Recherche et la Biodiversité (FRB, http://www.fondationbiodiversite.fr/) grant FRBCD-AOOI-07-12, the Action Concertée InterPasteurienne (ACIP) grant A-10-2009 and the ERA-Net BiodivERsA network (http://www.biodiversa.org/) with the national funders ANR France (ANR-13-EBID-0007-01), FWF Austria (FWF I-1437), and DFG Germany (DFG KL 2087/6-

Introduction

Human infectious diseases caused by vector-borne pathogens have an increasing incidence worldwide, accounting for 17% of the estimated burden of infectious diseases as referred by World's Health Organization [1]. Notably, arthropod-borne viruses (arboviruses) are emerging or re-emerging viruses transmitted to vertebrate hosts by the bite of infected arthropod vectors,

1), part of the 2012-2013 BiodivERsA call for research proposals.

Competing Interests: The authors have declared that no competing interests exist.

mainly mosquitoes. Among them, Chikungunya is a mosquito-borne viral infection caused by an alphavirus from the *Togaviridae* family. Chikungunya virus (CHIKV) is transmitted to humans by *Aedes* (*Stegomyia*) spp mosquitoes, primarily *Aedes aegypti*. Since 2004, CHIKV started a global spread with severe outbreaks in the Indian Ocean region, the Indian subcontinent and Central Africa, all associated with a single amino-acid change in the virus E1 glycoprotein that allowed an enhanced transmission by a secondary mosquito species, *Aedes albopictus* [2–4]. Autochthonous transmissions in Europe were also reported from Italy, with 217 confirmed cases in 2007 [5] and from France with two confirmed cases in 2010 [6]. Consecutively to a major chikungunya outbreak started in the French Antilles in 2013, autochthonous cases were reported in the United States [7] and more recently in France [8], bringing the threat of multiple outbreaks caused by virus-carrying travellers; in both temperate areas, *Ae. albopictus* was the vector responsible for CHIKV transmission.

The species *Ae. albopictus*, also known as tiger mosquito, is native from Southern and Eastern Asia but recently spread worldwide [9]. The rapid extension of *Ae. albopictus* combined with its ecological plasticity and vector competence for diverse arboviruses make the tiger mosquito a significant threat for public health [10]. In absence of effective vaccines or prophylaxis against most of arboviruses included CHIKV, current efforts are mainly based on controlling vector populations with insecticides. However, the development of mosquito resistance, as well as environmental contamination and side effects on non-target organisms has called chemical-based control methods into question [11]. Consequently, alternative and innovative vector control strategies emerged, and one of the most promising is based on the use of symbiotic bacteria [12]. In this framework, the endosymbiont *Wolbachia* has been the most studied candidate including arboviruses and parasites transmission control [13–15].

Wolbachia is an obligate intracellular bacterium that infects around 40% of arthropods [16], and manipulates their reproduction to facilitate its own spread among populations [17]. When the *w*Mel strain of *Wolbachia*, originated from *Drosophila*, was transinfected into *Ae. aegypti* embryos, mosquitoes presented limited vector competence for a large panel of pathogens including dengue virus (DENV) [18,19], CHIKV [19], yellow fever (YFV) [20], West-Nile virus (WNV) [21] and *Plasmodium* parasite [14,19]. However, it appears that *Wolbachia*-transinfected mosquitoes are markedly associated with a viral inhibition phenotype compared to naturally infected populations, which most of time exhibit no inhibition or even an enhancing of the infection [22]. In the field, *Ae. aegypti* lacks this association with *Wolbachia* while *Ae. albopictus* mosquitoes naturally carry two strains, namely *w*AlbA and *w*AlbB [23,24]. The native *Wolbachia* from *Ae. albopictus* was associated with a decrease of DENV transmission in mosquitoes from La Réunion island [25]. However, this phenotype was shown to be dependent on the mosquito population considered as no inhibition was observed in population from Houston, Texas [18]. Intriguingly, no significant impact of *Wolbachia* was observed on CHIKV transmission in *Ae. albopictus* population from La Réunion [26]. This suggests that the *Wolbachia* inhibition phenotype also depends on the viral strain considered. Together, these observations clearly indicate that the tripartite interaction between *Wolbachia*, arboviruses and their mosquito host is complex and varies according to the nature of the interacting partners.

The molecular and cellular mechanisms of *Wolbachia*-mediated inhibition of arboviruses are poorly known, but current hypotheses suggest a competition for host cell resources, supported by the bacterial density-dependent interference and the intra-host competition for amino acids and cholesterol [27,28]. Insect immune pathways activated upon *Wolbachia* infection have been also suggested to mediate the blocking phenotype, like autophagy [29], oxidative stress [30] or miRNA pathway [31]. It appears that *Wolbachia*-mediated activation of the Toll and Imd immune pathways was unlikely to trigger antiviral interference, as suggested by a recent study in *Drosophila* [32]. In addition, as being an obligate intracellular bacterium,

studies on *Wolbachia* are difficult using standard techniques. Interestingly, *Wolbachia*-infected cell lines were used as a tool to study the mechanisms involved in *Wolbachia*-pathogen interaction [29,30,33–35]. To facilitate the understanding of the *Wolbachia*/CHIKV/*Ae. albopictus* interaction, we built a cellular model by culturing the *w*AlbB strain *in vitro* into the *Ae. albopictus* CHIKV-permissive cell line C6/36. Using this simplified *in vitro* model, we measured the viral dynamic in the presence or absence of *Wolbachia*, and tried to decipher at which step of the viral cycle *Wolbachia* interferes with CHIKV infection. More broadly, this work provides a suitable tool to study *Wolbachia*-arbovirus interaction at the cellular level.

Material and Methods

Establishment of *Wolbachia*-infected mosquito cell line

The C6/36 cells, derived from *Ae. albopictus* larvae and originally non-infected by *Wolbachia*, were used for culturing *w*AlbB strain. This bacterial strain originated from naturally infected Aa23 cells isolated from *Ae. albopictus* eggs [36]. Both cell types were cultured at 28°C in growth medium consisting of equal volumes of Mitsuhashi/Maramorosh (Bioconcept, Switzerland) and Schneider's insect medium (Sigma, France) supplemented with 10% (v/v) of heat-inactivated foetal bovine serum (PAA, USA) and penicillin/streptomycin (50 U/50 μg/mL; Gibco, Invitrogen, France). Briefly, three 25 cm^2 flasks of confluent Aa23 cells were scrapped, pelleted for 10 min at 300×g and crushed by vortexing 10 min with 5-mm diameter sterile borosilicate beads (Biospec, OK, USA). Cell lysates were centrifuged for 5 min at 300×g, and supernatants were filtered through a 5-μM syringe filter (Millipore) to eliminate cellular debris. Fresh filtrate (500 μL) containing bacteria was inoculated onto 80% confluent monolayer of C6/36 cells, in shell vial tube (Sterilin, UK). After centrifugation 5 min at 2000×g, cells were incubated overnight at 28°C then the coverslip bearing cells was transferred into a 25 cm^2 flask with fresh culture medium and incubation period extended to reach 80% confluence. After this first round of infection, cells were harvested, resuspended in 500 μL of fresh medium and used for a second infection procedure. The *Wolbachia* infection in cells was characterized using electron microscopy, Fluorescent *In Situ* Hybridization (FISH) and quantitative PCR (qPCR). For each assay, we used as control tetracycline-treated cells (TET) to remove bacteria without modifying the host cell genetic background. This was achieved by adding 10 μg/mL of tetracycline hydrochloride (Sigma, France) in culture media of *Wolbachia*-infected (*w*AlbB) cells for 5 passages, and then cells were maintained in culture without tetracycline until use. The original C6/36 uninfected (CTRL), TET and *w*AlbB infected cells were continuously passaged in 25 cm^2 flasks by scrapping and seeding a new flask with 1:5 of the cell suspension in 5 mL of fresh medium, every 4 days.

Electron microscopy

The presence of *Wolbachia* in C6/36 cells was observed using electron microscopy at the Centre Technologique des Microstructures, University Lyon I (http://microscopies.univ-lyon1.fr/index.htm). Briefly, cells were washed in PBS twice and fixed in a 2% glutaraldehyde solution containing cacodylate buffer at pH 6.5, then postfixed in 1% osmium tetroxide in cacodylate buffer. Samples were then dehydrated in a graded series of ethanol and embedded in Epon. Ultrathin sections of 60 nm were performed using an UC7 ultramicrotome (Leica). After a contrast with uranyl acetate and lead citrate, the sections were observed using a Philips CM 120 Transmission Electron Microscope.

Virus

The CHIKV 06.21 strain derived from newborn serum sample with neonatal encephalopathy, was collected in La Reunion Island in 2005 [37]. This isolate was highly passaged in C6/36. Viral stocks were produced on C6/36 cells in 25-cm^2 flasks, at Multiplicity Of Infection (MOI) of 0.01. After 3 days at 28°C, supernatants from infected cells were recovered and virus titration was performed using plaque assay on Vero E6 (green monkey kidney) cells [38]. To measure the impact of tetracycline treatment on viral dynamics, CHIKV RNA titer was compared between CTRL and TET cells using quantitative RT-PCR (RT-qPCR), at two different MOI of 0.1 and 3. To that end, cells were transferred into 12-well plates at 1×10^6 cells per well and allowed to attach for 24 h, at 28°C. Infection with CHIKV 06.21 was performed in 2% FBS medium, using virus-free medium as control. After 1 h, 1.5 mL of fresh media with 10% FBS was added. Cells and supernatants were harvested at 2, 4, 6, 8, 10, 24, 48, 72, 96 and 168 hours post-infection. Residual cells were removed from supernatant by centrifugation for 3 min at full-speed and samples were stored at -80°C until titration. Adherent cells were rinsed twice in PBS and scrapped, pelleted by centrifugation and kept at -80°C prior to RNA isolation. Experiment was conducted with two independent samples. To assess the role of *Wolbachia* during CHIKV infection, we compared virus titer between TET and *w*AlbB bearing cells. The day prior infection, cells from three to six independent flasks were transferred in 12-well plates at 1×10^6 cells per well while another fraction was inoculated in shell vial tubes at 5×10^5 cells per tube for FISH staining. CHIKV 06.21 infection was performed as mentioned above, at MOI of 0.1 and 3, with cells and supernatant harvested at 1, 3, 5 and 7 days post-infection. Samples were stored at -80°C until use.

DNA and RNA isolation

Genomic DNA isolation was performed using *DNeasy blood and tissues* kit (Qiagen, France) following manufacturer's recommendations. After lysis in 180 μL of ATL buffer, samples were incubated for 2 h a 37°C with lysozyme (Euromedex, France) at a final concentration of 2 mg/mL. Residual co-extracted RNA was eliminated by adding 100 mg/mL of RNase A, for 2 min at room temperature. The isolated DNA was eluted in 30 μL of DNase-free water. Total RNA was isolated using the *RNeasy Mini Kit* (Qiagen, France) as recommended by supplier. Cell pellets were crushed in 350 μL RLT lysis buffer using RNase-free piston pellet (Kontes, USA), and RNA was eluted in 37 μL of RNase-free water. RNA solution was treated with DNase using the Ambion *TURBO-DNA free* kit (Ambion, USA) in 50 μL final volume following the manufacturer's instructions. DNA and RNA were quantified using a UV-mc^2 spectrophotometer and diluted to 5 ng/μL, then frozen at -20°C (DNA) or -80°C (RNA) until use.

Quantitative *Wolbachia* PCR analysis

The relative density of *Wolbachia* per cell was monitored by qPCR using *Wolbachia* Surface Protein (*wsp*) gene for the bacterium and *actin* gene for the host cell. Standard curves were drawn on 10-fold serial dilutions from 1×10^8 to 1×10^1 copies/μL of the DNA plasmid *pQuantAlb16S* containing *wsp* and *actin* gene fragments [23]. Each 20 μL reaction contained 10 ng (2 μL) of template DNA, 10 μL Fast-SYBR-Green Master Mix (Roche, Suisse), 200 mM (*wsp*) and 300 mM (*actin*) of primers (Table 1). Amplification was performed on LC480 LightCycler (Roche, France) and consisted of 10 min at 95°C, followed by 40 cycles of 15 s at 95°C, 1 min at 65°C, and a final elongation at 72°C for 30 s. All PCR reactions were done in triplicate and DNA from C6/36_TET was used as negative control.

Table 1. List of primers and probes used in this study.

Primers	Sequence (5'-3')	Reference
183F	AAGGAACCGAAGTTCATG	[77]
QBrev2	AGTTGTGAGTAAAGTCCC	[77]
actAlb-dir	GCAAACGTGGTATCCTGAC	[77]
actAlb-rev	GTCAGGAGAACTGGGTGCT	[77]
Chik/E2/9018/+	CACCGCCGCAACTACCG	[78]
Chik/E2/9235/–	GATTGGTGACCGCGGCA	[78]
**Oligonucleotide probes**		
W2	Rhodamine-CTTCTGTGAGTACCGTCATTATC	[79]
Wol3	Rhodamine-TCCTCTATCCTCTTTCAATC	[80]
Chiknsp2	Alexa488-CAAGTCAGCTATTATCAAGAACCTAGTTAC	*this study*
ChikE2	Alexa488-GAGATAATTCTGTATTATTATGAGCTGTAC	*this study*

doi:10.1371/journal.pone.0125066.t001

CHIKV RT-qPCR analysis

The CHIKV RNA copy number was quantified by RT-qPCR targeting the envelope *E2* gene. Viral RNA copies were assessed using a standard curve of 10-fold serial dilution of a synthetic CHIKV RNA transcript [26]. One-step RT-qPCR was performed using EXPRESS One-Step SYBR GreenER Kit (Invitrogen, France) in a volume of 20 μL containing 10 ng (2 μL) of RNA template, 10 μL EXPRESS SYBR GreenER SuperMix Universal, 200 nM of sense Chik/E2/9018/+ and anti-sense Chik/E2/9235/– primers (Table 1) and 0.5 μL EXPRESS Superscript Mix. Amplification was performed on a LC480 LightCycler (Roche, France) and consisted of 15 min at 50°C followed by 95°C for 2 min, then 40 cycles of 95°C for 15 s and 63°C for 1 min. All PCR reactions were performed in triplicate and RNA from CHIKV-uninfected C6/36 cells was used as negative control.

Fluorescent focus assay (FFA)

Virus infectious titer was quantified using an indirect immunofluorescent detection of infectious foci on C6/36 monolayer [39]. Cells were seeded in 96-well plates at a density of 3×10^6 cells/well and incubated for 36 h at 28°C to produce confluent monolayers. Ten-fold serial dilutions of sample supernatants were inoculated in a final volume of 50 μL/well. After 1 h incubation at 28°C to allow viral adsorption, with gently rocking every 15 min to spread viral inoculum, an overlay consisting of 5% FBS, 1.6% of carboxymethyl cellulose (CMC, VWR) was added in a final volume of 200 μL per well. Plates were incubated 3 days at 28°C then 150 μL of freshly prepared 4% formaldehyde solution in PBS was added without removing the overlay. Cell monolayers were fixed for 20 min at RT, washed three times in PBS, then incubate for 30 min at RT in PBS-0.1% Triton X-100. Plates were stained for 1 h at 37°C with a 1:1000 dilution of hyper-ascetic immune fluid specific to CHIKV 06.21 in PBS-0.1% Bovine Serum Albumin (BSA, Sigma, France). After 3 washes in PBS, cells were incubated for 1 h at 37°C with an anti-mouse Alexa488-conjugated antibody (Molecular probes, Invitrogen, France) diluted at 1:200 in PBS-0.1% BSA followed by three washes in PBS and a final wash in distilled water. Cell monolayers were observed using an EVOS inverted fluorescence microscope (Life Technologies, France) with a FITC-filter, under 10X objective. The total number of fluorescent foci was counted from 5 to 50 at the appropriate dilution, and virus titer was calculated as fluorescent focus unit per mL. The titer represents a mean of two independent samples.

Fluorescent *In Situ* Hybridization

After two washes in PBS, cells were fixed on the coverslip for 10 min in freshly prepared 4% formaldehyde in PBS. Hybridization was conducted overnight at 37°C in 1 mL of hybridization buffer [formamide 50%, SSC (saline-sodium citrate) 5X, 200 mg dextran sulfate per mL and 250 μg poly(A) per mL, 250 μg salmon sperm DNA per mL, 250 μg tRNA per mL, DTT (1,4-dithiothreitol) 0.1 mg/L, Denhartdt's solution 0.5X] containing 200 ng of *Wolbachi*a probes W2 and Wol3 labelled in their 5'-end with Rhodamine Red-X and CHIKV probe labelled in 5'-end with Alexa488 fluor (Table 1). After hybridization, samples were washed twice in 1X SSC-10 mmol/L DTT and then twice in 0.5X SSC-10 mmol/L DTT at 55°C for 15 min each. Cells were then rinsed in PBS, mounted on a glass slide with 3 μL of DAPI (4',6-diamidino-2-phenylindole, dihydrochloride) solution (1 μg/mL of dye) in glycerol/PBS (1:1). Samples were viewed under a fluorescence microscope (AXIO Imager.ZI; Zeiss, France). To estimate the proportion of cells infected by *Wolbachia*, five different microscope fields were analyzed with at least 50 cells per field [40].

Statistics

The continuous response variables (viral and bacterial titers) were $\log_{10}$-transformed. They were analysed using a multifactorial linear model, with a normal error distribution and an identity link function that included the effect of the time and MOI as ordinal variables, treatment as discrete variable and their interactions. All the statistical analysis was performed using R environment (version 3.1.0).

Results

Characterization of *w*AlbB infection in mosquito cells

Previous studies mentioned that the *w*AlbB strain could be maintained in C6/36 [41,42]. Despite this, *w*AlbB dynamics of infection in C6/36 remains unknown. The *w*AlbB cells were purified from Aa23 cells, as they were already adapted to cell line culture. The C6/36 cells tend to grow in adhesive cell clusters, forming patchy monolayers independently of *Wolbachia* infection (S1 Fig). Two attempts were necessary to obtain *Wolbachia* infected cells, designated C6/36_*w*AlbB, with a *wsp* signal in PCR persisting in cells after several passages (not shown). Electron microscopy of C6/36_*w*AlbB cells (P.30) revealed the presence of *Wolbachia* as round-shaped particles of varying size inside the cytoplasm, surrounded by a host cell membrane where the bacteria seem to divide (Fig 1). As expected, no *Wolbachia* was seen outside a cell, while some bacteria could be released after the lysis of their host cell. In C6/36_TET cells, i.e. cells cured from *Wolbachia* by tetracycline treatment, no difference in cell aspect was noted compared to *Wolbachia*-infected cells, despite the absence of *Wolbachia* infection. The C6/36_*w*AlbB cells were maintained in continuous culture for 40 passages, corresponding to approximately 5 months. Quantitative PCR analysis showed that the density of *Wolbachia* was highly dynamic according to the passages (Fig 2), with the lowest density of 0.9 *wsp*/*actin* ratio at P.7 to 67.6 *wsp*/*actin* ratio at P.17 for the highest. After P.17, *Wolbachia*'s density decreased to remain around 10 *wsp*/*actin* ratio from P.36 to P.40. The C6/36_TET cells were negative for *Wolbachia* infection in qPCR. The FISH also confirmed the absence of *Wolbachia* in C6/36_TET cells whereas the bacteria were detected in C6/36_*w*AlbB cytoplasm (Fig 3A), even if the infection did not reach 100% of the cultured cells (Fig 3B). Along with the density of bacteria measured in qPCR, the *Wolbachia* fluorescent signal decreased from P.15 to P.37 and that goes together with a significantly lower proportion of *Wolbachia*-infected cells from 92.4% to 45.3% at P.15 and P.37, respectively ($P < 2.2e^{-16}$) (Fig 3B).

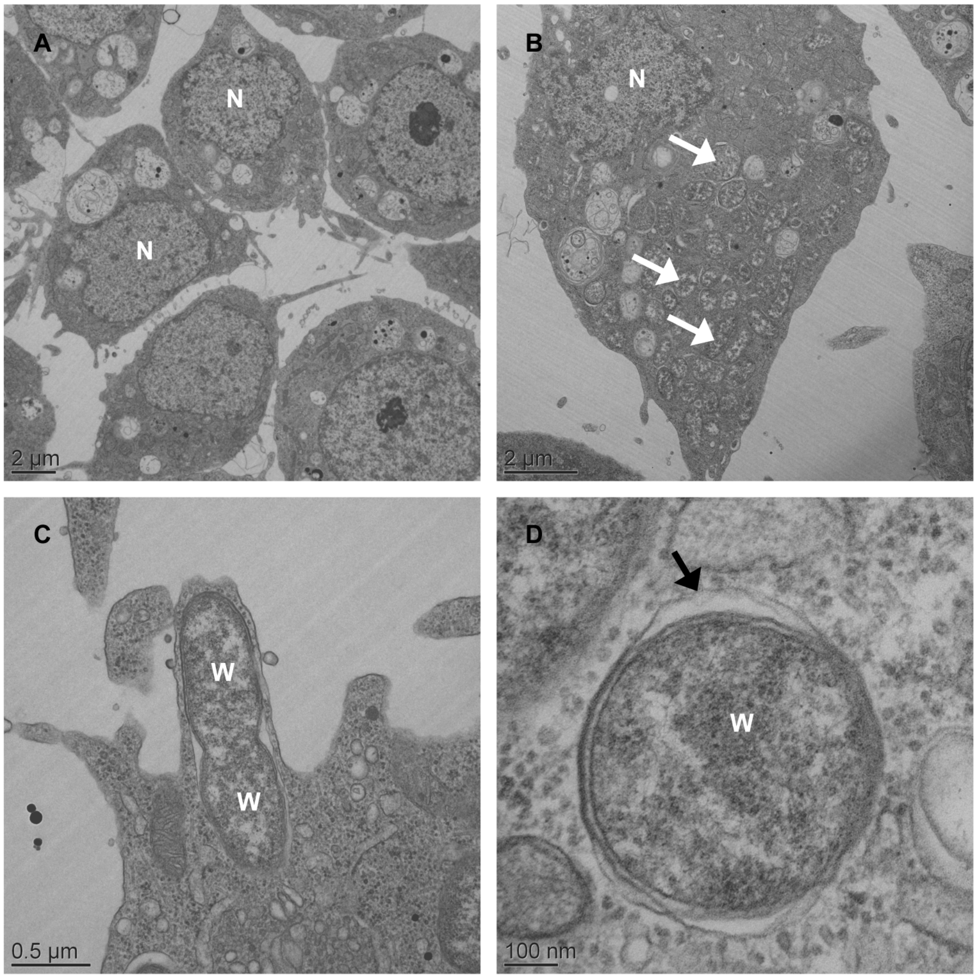

Fig 1. Electron microscopy of *Wolbachia* in *Aedes albopictus* C6/36 cells. Low-magnification transmission electron micrograph of C6/36_TET cells with no bacterial signal in host cell cytoplasm (**A**) whereas *Wolbachia* (white arrowhead) are seen throughout the cytoplasm of C6/36_*w*AlbB cells (**B**). *Wolbachia* presumably is undergoing the process of cell division (**C**). High-magnification micrograph of *Wolbachia* in cytoplasm of the host cell showing a membranous structure surrounding the bacterium (black arrowhead) (**D**).

doi:10.1371/journal.pone.0125066.g001

Reduced CHIKV infection by *w*AlbB *in vitro*

As no viral inhibition was measured for CHIKV 06.21 in orally infected *Ae*. *albopictus* mosquitoes [26], we tested the interaction of *w*AlbB and CHIKV 06.21 in C6/36. First, we assessed that CHIKV replication was not affected by anti-*Wolbachia* tetracycline treatment, as viral RNA titer was not significantly different between C6/36_TET and C6/36_CTRL cells at MOIs of 0.1 ($P = 0.45$) and 3 ($P = 0.68$) (Fig 4). The viral RNA titer increased from 2 h to 72 h post-infection (pi), with a short eclipse phase between 8 h and 10 h pi, then decreased until 96 h to reach a plateau until day 7 pi. The viral replication was dramatically reduced in C6/36_*w*AlbB compared to C6/36_TET cells as measured by RT-qPCR after infection at MOI 0.1 (Fig 5A). The RNA titer significantly decreased in C6/36_*w*AlbB cells by at least ten-fold across all time-points. Interestingly, *Wolbachia*-mediated inhibition depended on the time of infection (*Wolbachia**time interaction, $P<2E\text{-}16$), suggesting that presence of *Wolbachia* could delay virus replication as previously mentioned [43]. Although viral RNA titer decreased, inhibition was

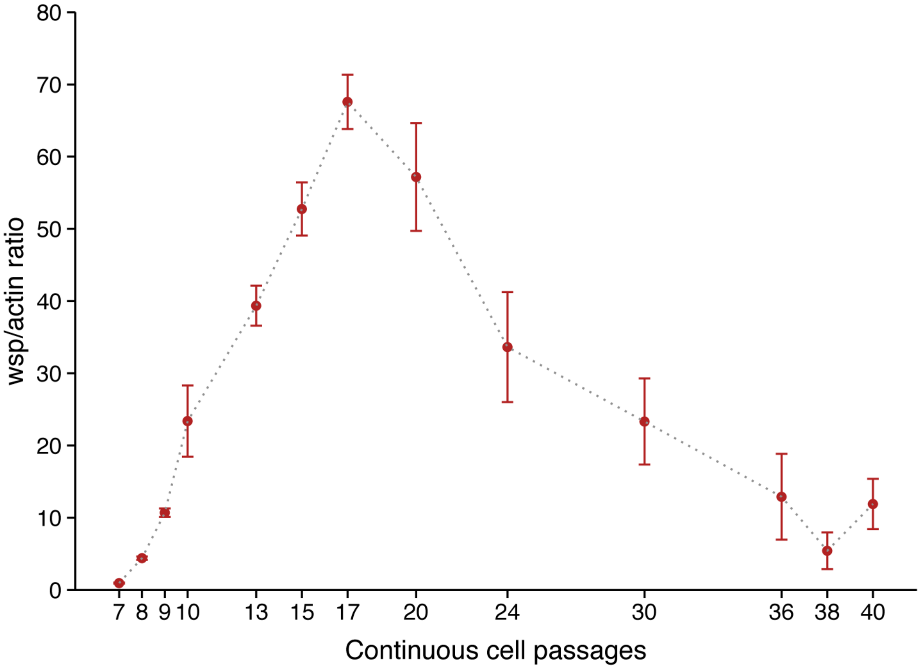

Fig 2. Dynamics of *w*AlbB infection in C6/36 cells. Ratio of *Wolbachia wsp* copies per host *actin* copies during continuous cell culture, measured by qPCR on total genomic DNA (error bars represent the standard deviation of the mean of three independent samples).

doi:10.1371/journal.pone.0125066.g002

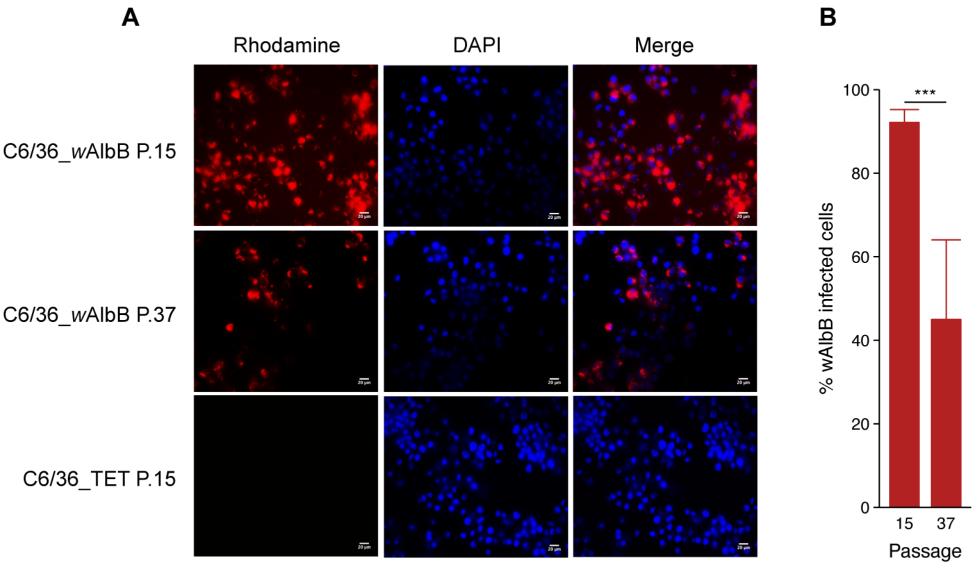

Fig 3. Proportion of *Wolbachia*-infected cells detected by Fluorescence *in situ* Hybridization. Rhodamine-labelled oligonucleotide probe designed on *Wolbachia* 16S rRNA gene (red) detected the bacteria in the cytoplasm of the host cell at passages P.15 and P.37 (**A**). Nuclei of the host cells are shown in blue after DAPI labelling (bars = 20μm). Percentage of cells with a *Wolbachia*-positive signal in FISH at P.15 and P.37 (**B**) (Error bars represent the standard deviation of the mean of 50 independent microscope fields from three independent samples).

doi:10.1371/journal.pone.0125066.g003

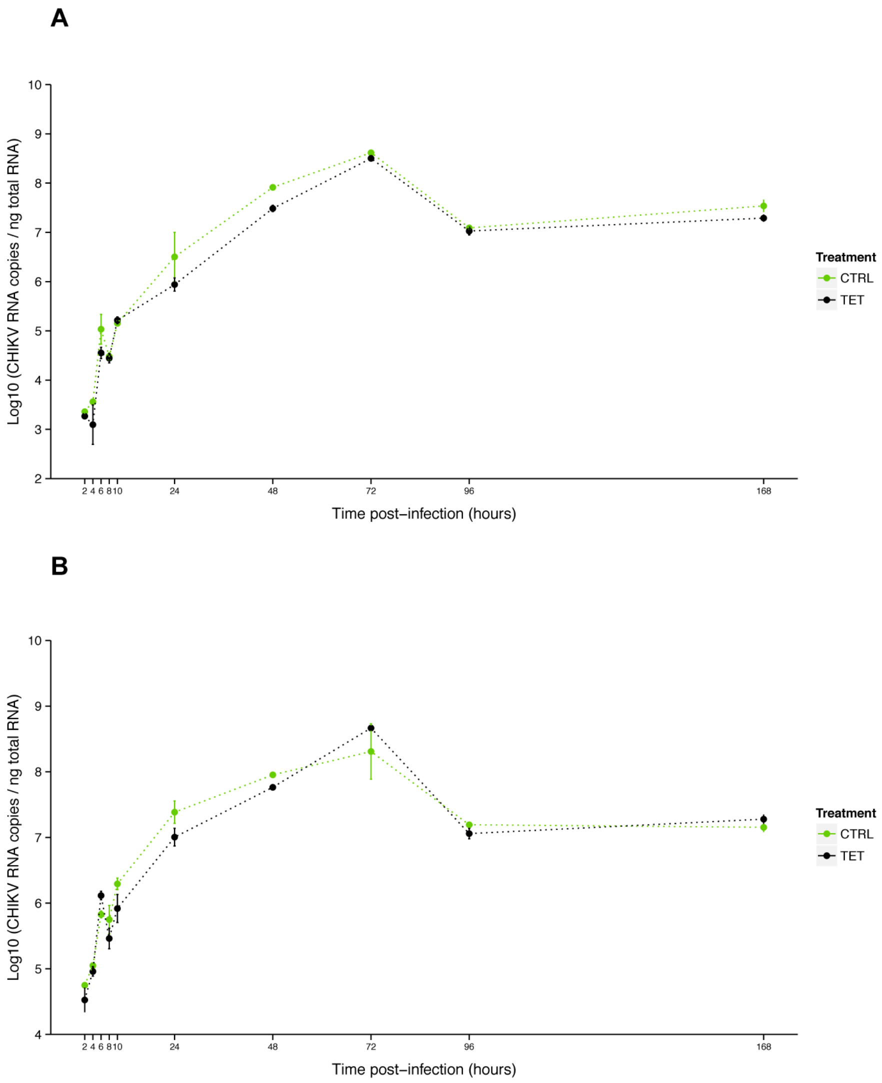

Fig 4. Effect of tetracycline treatment on CHIKV growth in C6/36. Kinetics of CHIKV RNA titer at MOI 0.1 (**A**) and 3 (**B**) measured by RT-qPCR on total cellular RNA isolated from C6/36 cells (non infected by *Wolbachia*) treated with tetracycline (TET) or not (CTRL). Error bars represent the standard deviation of the mean of two independent samples.

doi:10.1371/journal.pone.0125066.g004

not complete with at least 4.81 $\log_{10}$ CHIKV RNA copies per ng total RNA in C6/36_*w*AlbB cells at day 1 pi, where *Wolbachia* antiviral effect seemed to be the strongest. CHIKV inhibition by *w*AlbB was also measured at the RNA infectious particles level using FFA assay on cell supernatants (Fig 5B). A major decrease of viral infectious titer was detected in C6/36_*w*AlbB compared to C6/36_TET cells, depending on the time post-infection (*Wolbachia**time interaction, $P = 0.00177$). As for viral RNA, this suggests that *Wolbachia*-mediated inhibition of viral infectious particles production decreases with the time of infection, even if the time effect is lower than for viral RNA decrease. The *w*AlbB density was monitored in both CHIKV infected (CHIKV+) and uninfected (CHIKV-) cells using qPCR (Fig 6). The bacterial load did not vary according to viral infection ($P = 0.228$) but time had a significant effect (P<2E-16). The *Wolbachia* titer increased with time, ranging from 13.3 to 25.7 *wsp*/*actin* ratio at day 1 and 7 pi, respectively.

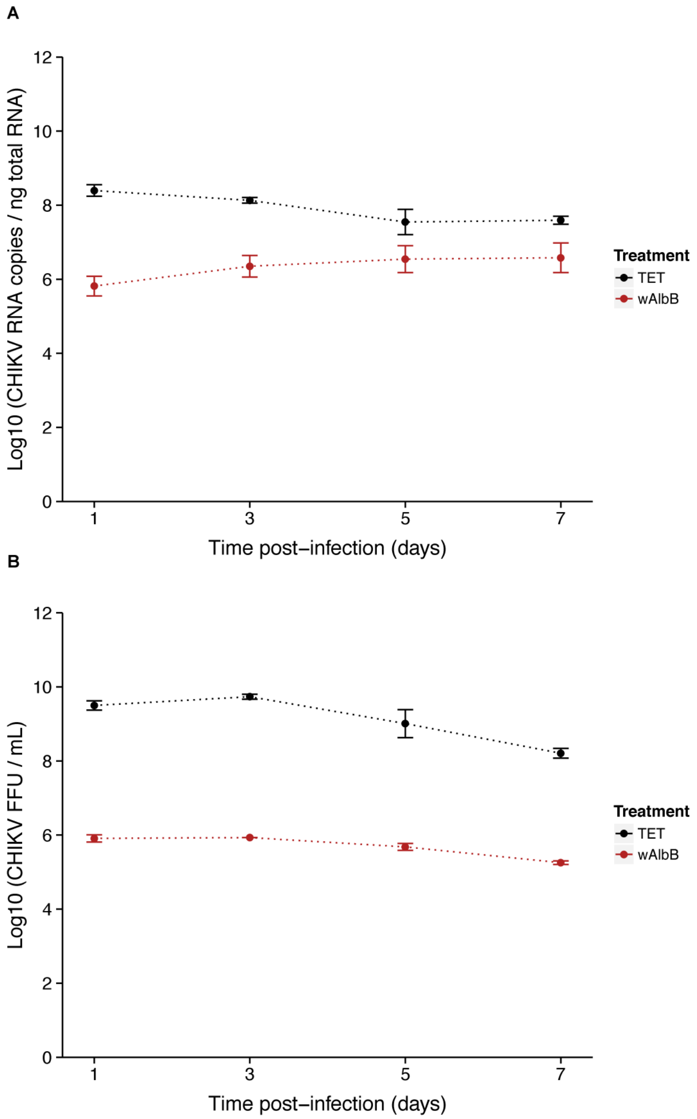

Fig 5. Effect of *Wolbachia* on CHIKV replication and infectiosity. Kinetics at MOI 0.1 of CHIKV RNA titer measured by RT-qPCR on total cellular RNA (**A**) and CHIKV infectious titer in supernatant measured by FFA (**B**) in presence of *Wolbachia* (wAlbB) or in cells cured from the bacteria by tetracycline treatment (TET). Error bars represent the standard deviation of the mean of three independent samples.

doi:10.1371/journal.pone.0125066.g005

CHIKV infection of *w*AlbB-colonized cells

The FISH technique was shown to be an efficient method to detect viruses in mosquito cells [44]. This is the first time such a technique was used to detect CHIKV. The oligonucleotide-probes designed can also detect other alphaviruses, namely Sindbis virus and Ross River virus (not shown). The results showed that CHIKV could be labelled in the cytoplasm of infected cells whereas no CHIKV signal was detected in uninfected cells (Fig 7). Moreover, viral RNA was also detected in cells previously infected with *Wolbachia*, indicating that at least in some cells the virus is able to penetrate in spite of the presence of the bacterium. However, the co-localization of both *Wolbachia* and CHIKV was not detected in many cells, and the use of

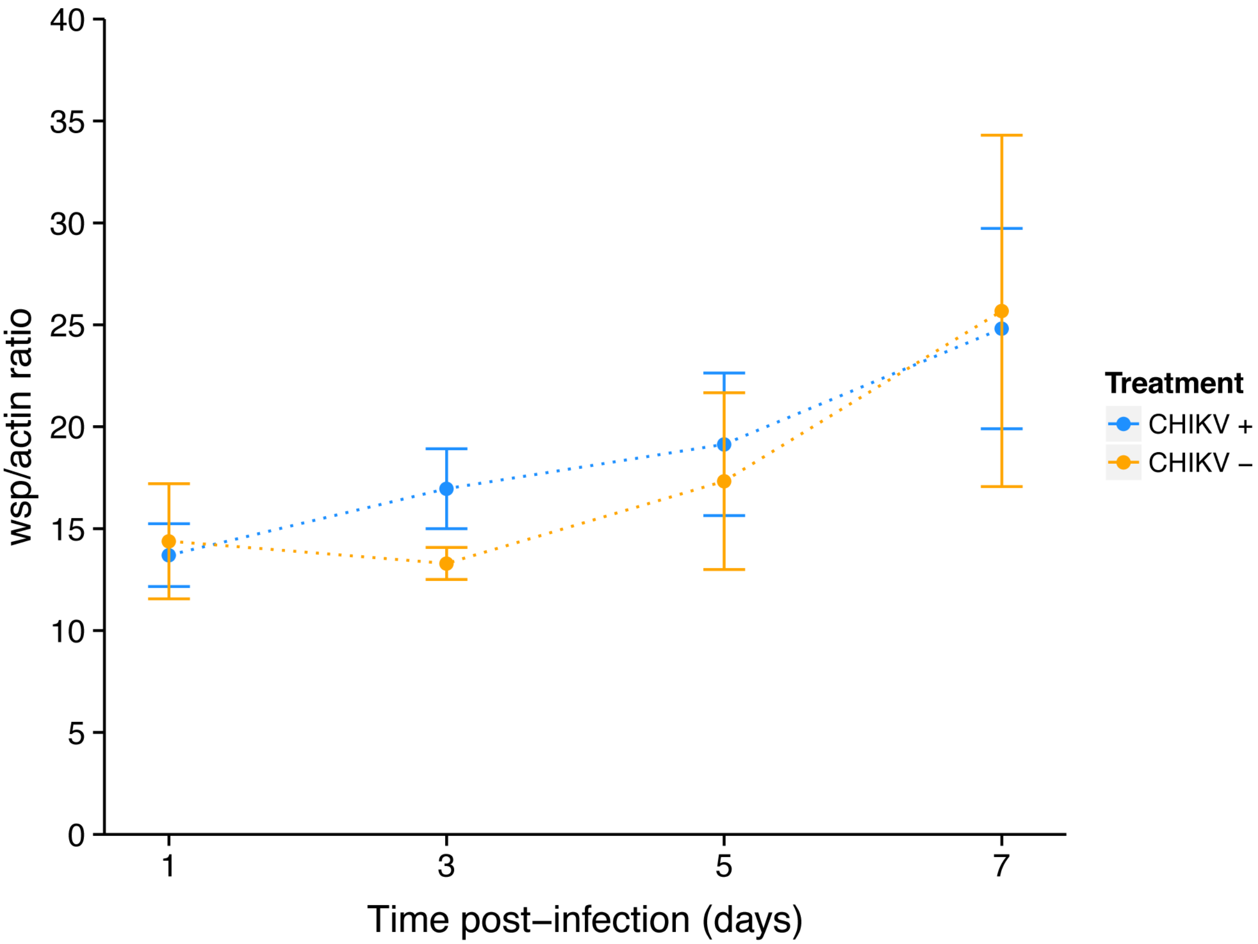

Fig 6. Dynamics of *w*AlbB in C6/36 during CHIKV infection. Ratio of *Wolbachia wsp* copies per host *actin* copies during CHIKV infection at MOI 0.1, measured by qPCR on genomic DNA. Error bars represent the standard deviation of the mean of three independent samples.

doi:10.1371/journal.pone.0125066.g006

FISH technique did not allowed us to tell if the presence of both micro-organisms in the same host cell was correlated with the load of either bacterium or virus.

Discussion

Ae. albopictus is naturally infected by *Wolbachia* and remains an important vector of CHIKV [45,46] and in a lesser extent of DENV [47,48]. Intriguingly, the pattern of *Wolbachia*-arbovirus interaction in *Ae. albopictus* remains unclear. Previous studies showed that transinfection of *Ae. albopictus* with the *w*Mel strain of *Wolbachia* is likely to induce DENV and CHIKV inhibition [49,50]. However, *Ae. albopictus* is naturally co-infected with *Wolbachia w*AlbA and *w*AlbB strains but no blocking phenotype was measured against DENV and CHIKV in populations from Houston [18,51] and La Réunion [26], respectively. Conversely, a decrease of DENV titer was observed in the saliva of symbiotic females in the *Ae. albopictus* population from La Réunion [25]. This suggests that *Wolbachia*'s potential to interact with viral replication in its native mosquito host depends on the combination of bacterial strain, vector and virus factors thus making the study of this multipartite interaction very complex. Therefore, simplified models are needed to explore *Wolbachia*-pathogen interaction in mosquito. As *Wolbachia* is an obligate intracellular bacterium, insect cell lines have been widely used for culturing the

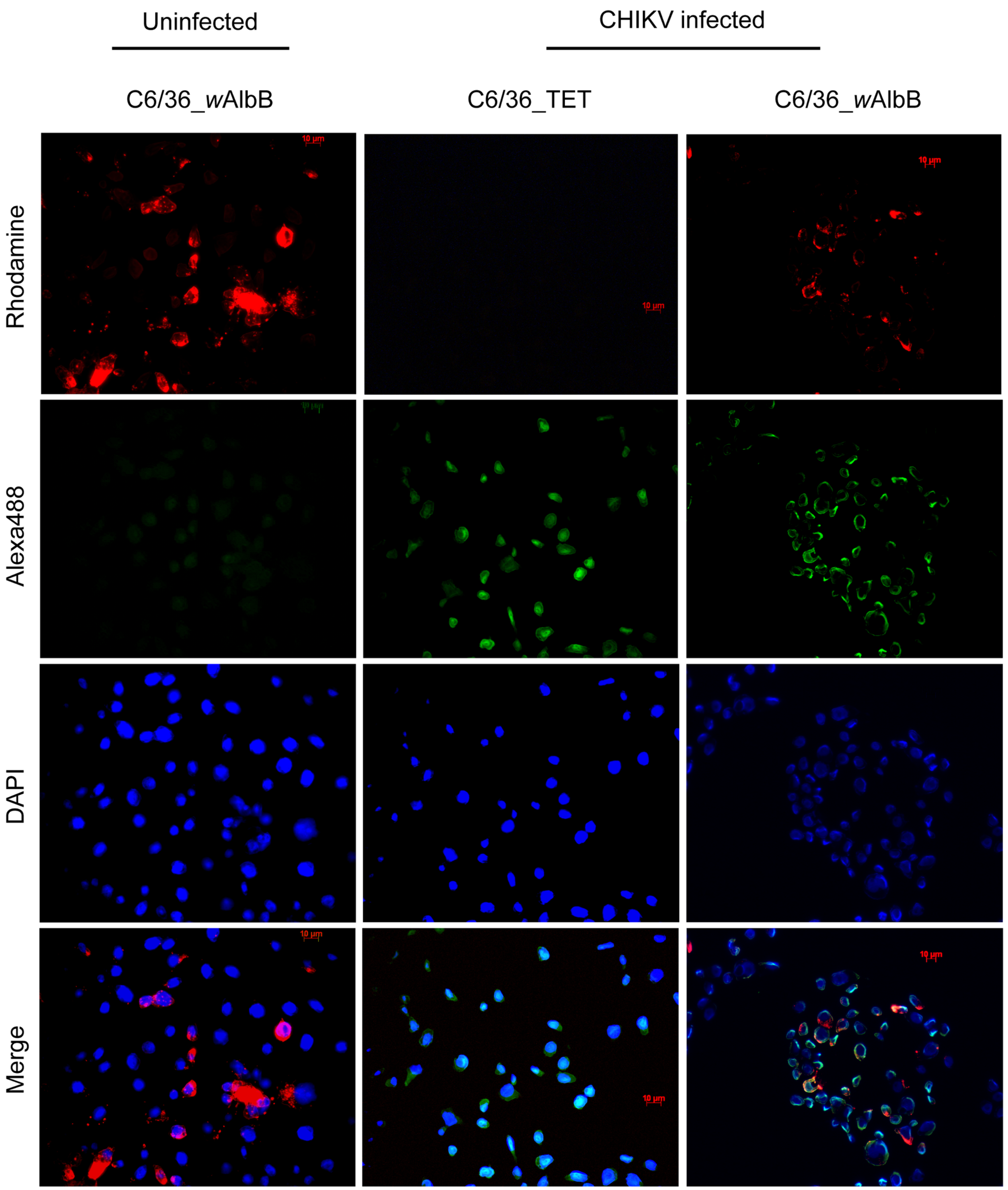

Fig 7. Detection and localization of *w*AlbB and CHIKV *in cellulo* by FISH. Detection of *Wolbachia* 16S rRNA gene (red) and CHIKV *Env* RNA (green) using oligonucleotide probes labelled with Rhodamine and Alexa488, respectively. *Wolbachia* signal is detected in C6/36_*w*AlbB but not in tetracycline treated cells (C6/36_TET). CHIKV signal is detected only in CHIKV infected modality, in the absence or in the presence of *Wolbachia* where it co-localize with the bacteria in the cytoplasm of C6/36_*w*AlbB cells. Nuclei of host cells are shown in blue after DAPI labelling (bars = 10 µm).

doi:10.1371/journal.pone.0125066.g007

bacterium with special emphasis on *Ae. albopictus* derived cells [29,36,41,42,52–55]. It is worth noting that previous studies showed a high discrepancy in *Wolbachia* density in cell culture [42,55,56], possibly due to cells passaging method that could result in reduction or loss of bacterial infection [55]. The initial burst of infection followed by a rapid decrease of *w*AlbB density in C6/36 could be interpreted as an adaptation of the bacterium to cell culture as previous work noticed a shift in *Wolbachia* phenotype in its natural host after long-term passage in mosquito cell line [52]. It would be worth to study the mechanisms underpinning *Wolbachia* establishment in mosquito cell line and explore possible correlation with antiviral activity. Moreover, mosquito cell lines are generally permissive to arbovirus infection, providing a useful tool to study *Wolbachia*-arbovirus interaction at a finer scale [21,35,51]. In adult mosquito, during the Extrinsic Incubation Period (EIP), the virus infects essentially somatic tissues including midgut and salivary glands [57] which are both infected by *Wolbachia* in *Ae. albopictus* [23]. C6/36 cells, which originated from uninfected somatic tissue, appear to be an appropriate model in complement to Aa23 cells to study *Wolbachia*-arbovirus interaction in an *Ae. albopictus* background.

Previous studies suggested that DENV inhibition seems to depend on *Wolbachia* density [51,58]. We showed that in C6/36, *w*AlbB density is highly dynamic but remains low compared to Aa23 with a maximum at 72.5 *wsp*/*actin* copies against 1,888.3 *wsp*/*actin*, respectively [51]. However, we observed a significant CHIKV interference in C6/36_*w*AlbB at a relative *Wolbachia* density of 13.7 to 25.6 *wsp*/*actin*, although inhibition was not complete. These results suggest that *Wolbachia*-mediated antiviral activity can occur *in vitro* even at low bacterial density. Interestingly, Lu and colleagues extrapolated from their observations in Aa23_*w*AlbB cells that a relative density of *w*AlbB of 0.3, 5.3, and 12.3 *wsp*/*actin* in midgut, salivary gland and fat body of *Ae. albopictus*, respectively was too low to interfere with DENV infection *in vivo* [51]. The lower abundance of *w*AlbB in *Ae. albopictus* organs compared to C6/36 cells [23] is in line with this observation, and with the absence of CHIKV inhibition measured *in vivo* in *Ae. albopictus*. Conversely, the viral load did not seem to counteract with virus blocking by *Wolbachia* as demonstrated in C6/36_*w*MelPop-CLA cells infected with DENV [35]. Using the *Ae. aegypti* cell line Aag-2 to culture *w*MelPop-CLA, it was recently shown that *Wolbachia*-induced antiviral activity occurred as soon as the RNA replication step for DENV, but only at the step of virion assembly/secretion for WNV [21]. These results emphasize the importance of measuring both RNA and infectious particles to assess *Wolbachia*-antiviral activity, and suggest that distinct antiviral cellular mechanisms are involved during *Wolbachia*-virus interaction. In our model, CHIKV replication is inhibited by *w*AlbB in C6/36 cells, in a time-dependent manner with the lowest viral RNA load measured at 24 h pi. We also observed a decrease of infectious particles titer in supernatant as early as 24 h pi, indicating that viral blocking could occur at both stages of the viral cycle. This also suggests that CHIKV blocking by *w*AlbB could occur at the early stage of viral infection. Considering this, FISH was used to label both *Wolbachia* and CHIKV during co-infection of C6/36 cells. The FISH experiment showed that *Wolbachia* and CHIKV could be localized in the same host cell, indicating that *w*AlbB did not seem to inhibit CHIKV infection by preventing viral entry, at least in some cells. This hypothesis is reinforced by *in vivo* confocal microscopy where *Wolbachia* was co-localized with DENV in *Ae. albopictus* salivary glands [25] as well in *Ae. aegypti* tissues, where detection by FISH supported a cellular exclusion of DENV by the *w*Mel strain of *Wolbachia* [19]. However, even if *Wolbachia*-virus co-infected cells or tissues are detected in *Ae. albopictus* both *in vitro* and *in vivo*, their magnitude cannot exclude that viruses preferentially infect *Wolbachia*-free compartment.

The cellular pathways involved during virus blocking by *Wolbachia* are poorly known and indirect effects were mentioned to explain *Wolbachia*-mediated antiviral phenotype. The mechanisms of antiviral response in insects relies on different innate immune pathways, the

main one being the small interfering RNA (siRNA) pathway [59]. It was recently shown that C6/36 lacks a functional siRNA mechanism [60], suggesting that siRNA pathway is not involved in *w*AlbB-mediated CHIKV interference. *Wolbachia* was shown to manipulate another RNA interference pathway, the micro-RNA (miRNA) pathway, to facilitate its own spread in the mosquito, and this mechanism could be involved in DENV interference [61,62]. It has been proposed that *Wolbachia*-induced antiviral phenotype relies through the activation of mosquito innate immune system, including Imd and Toll pathways [19]. However, a recent study using *Drosophila* mutant's deficient for Toll and Imd genes conclude that neither is required for the bacteria to inhibit DENV [32]. In the meantime, it has been suggested that *Wolbachia* and the virus could engage a direct competition for host cell resources, as underlined by the importance of host cholesterol levels for *Drosophila* C virus blocking in *D. melanogaster* [27]. We demonstrated in previous work that *w*Mel manipulates iron metabolism in *Ae. albopictus* RML-12 cells through bacterioferritin expression [63], another potential explanation for its antiviral activity as iron load is involved in the modulation of innate immunity [64]. Further unexplored hypothesis is autophagy, a mechanism that has been shown recently to regulate *Wolbachia* density across different arthropod hosts including mosquito cells [29]. The autophagy pathway is required by CHIKV to replicate [65], and this cellular function could be involved in *Wolbachia* antiviral interference.

Overall, insect cell lines may represent a promising tool to facilitate the understanding of *Wolbachia*-pathogen interaction notably through electron microscopic observations of cell structural changes, and transcriptomic or proteomic studies which could allow to identify host infection regulatory pathways influenced by *Wolbachia* [34,66–68]. The potential direct activity of *Wolbachia* derived compounds against pathogens remains unknown but need further exploration, especially in the light of recent results suggesting the direct anti-DENV activity of a *Chromobacterium* sp (*Csp_P*) isolated from *A. aegypti* midgut [69]. Our results showed a significant antiviral effect of *w*AlbB against CHIKV *in cellulo* that was not measured *in vivo* at the mosquito organ level, even if CHIKV RNA load was constraint in a smaller range in symbiotic females organs [26]. This emphasizes the need to better understand *Wolbachia* symbiosis in its native host *Ae. albopictus*, and its impact on vector competence [70]. Mosquito vector competence for arboviruses depends on multiple factors such as mosquito genotype, virus genotype and their interaction [71] but also temperature [72,73] or mosquito microbiota [74]. Recent studies showed that pathogen blocking by *Wolbachia* was influenced by temperature [75] and that bacteria from the genus *Asaia* can inhibit vertical transmission of *Wolbachia* in *An. gambiae* [76]. Together, these results underline the importance of exploring *Wolbachia*-pathogen interaction, especially in a context where *Wolbachia*-infected mosquitoes represent a promising strategy to control vector-borne diseases.

Supporting Information

S1 Fig. C6/36_*w*AlbB cells in transmission-light microscopy. Pictures in light microscopy of C6/36 cells infected by *Wolbachia* (C6/36_*w*AlbB) or tetracycline-treated (C6/36_TET) during their growth in F25 cm^2 flasks, between two passages (bars = 20 µm).
(TIF)

Acknowledgments

We thank Karima Zouache and Aurélien Mercier for providing mouse hyper-ascetic immune fluid against CHIKV 06.21, as well as Denis Voronin and Louis Lambrechts for advices in FISH and in statistical analysis, respectively. We acknowledge the contribution of the BSL3

platform of SFR BioSciences Gerland Lyon Sud (UMS3444/US8) as well as the DTAMB platform of the FR41 Bio-Environment and Health (University Lyon 1) and the Centre Technologique des Microstructures (University Lyon 1). This work was carried out within the framework of Groupement de Recherche International (GDRI) Biodiversity and Infectious Diseases and Biodiversité et Développement Durable.

Author Contributions

Conceived and designed the experiments: VR CVM PM. Performed the experiments: VR CVM YS FHT. Analyzed the data: VR CVM PM. Contributed reagents/materials/analysis tools: PP PM. Wrote the paper: VR CVM PM.

References

1. WHO | About vector-borne diseases. In: WHO [Internet]. [cited 15 Nov 2014]. Available: http://www.who.int/campaigns/world-health-day/2014/vector-borne-diseases/en/
2. Tsetsarkin KA, Vanlandingham DL, McGee CE, Higgs S. A single mutation in chikungunya virus affects vector specificity and epidemic potential. PLoS Pathog. 2007; 3: e201. doi: 10.1371/journal.ppat.0030201 PMID: 18069894
3. Tsetsarkin KA, Weaver SC. Sequential adaptive mutations enhance efficient vector switching by Chikungunya virus and its epidemic emergence. PLoS Pathog. 2011; 7: e1002412. doi: 10.1371/journal.ppat.1002412 PMID: 22174678
4. De Lamballerie X, Leroy E, Charrel RN, Ttsetsarkin K, Higgs S, Gould EA. Chikungunya virus adapts to tiger mosquito via evolutionary convergence: a sign of things to come? Virol J. 2008; 5: 33. doi: 10.1186/1743-422X-5-33 PMID: 18304328
5. Angelini R, Finarelli AC, Angelini P, Po C, Petropulacos K, Silvi G, et al. Chikungunya in north-eastern Italy: a summing up of the outbreak. Euro Surveill Bull Eur Sur Mal Transm Eur Commun Dis Bull. 2007; 12: E071122.2.
6. La Ruche G, Souarès Y, Armengaud A, Peloux-Petiot F, Delaunay P, Desprès P, et al. First two autochthonous dengue virus infections in metropolitan France, September 2010. Euro Surveill Bull Eur Sur Mal Transm Eur Commun Dis Bull. 2010; 15: 19676.
7. Leparc-Goffart I, Nougairede A, Cassadou S, Prat C, de Lamballerie X. Chikungunya in the Americas. Lancet. 2014; 383: 514. doi: 10.1016/S0140-6736(14)60185-9 PMID: 24506907
8. WHO | Chikungunya–France. In: WHO [Internet]. [cited 15 Nov 2014]. Available: http://www.who.int/csr/don/23-october-2014-chikungunya/en/
9. Bonizzoni M, Gasperi G, Chen X, James AA. The invasive mosquito species *Aedes albopictus*: current knowledge and future perspectives. Trends Parasitol. 2013; 29: 460–468. doi: 10.1016/j.pt.2013.07.003 PMID: 23916878
10. Benedict MQ, Levine RS, Hawley WA, Lounibos LP. Spread of the tiger: global risk of invasion by the mosquito *Aedes albopictus*. Vector Borne Zoonotic Dis Larchmt N. 2007; 7: 76–85. doi: 10.1089/vbz.2006.0562 PMID: 17417960
11. David J-P, Coissac E, Melodelima C, Poupardin R, Riaz MA, Chandor-Proust A, et al. Transcriptome response to pollutants and insecticides in the dengue vector *Aedes aegypti* using next-generation sequencing technology. BMC Genomics. 2010; 11: 216. doi: 10.1186/1471-2164-11-216 PMID: 20356352
12. Christodoulou M. Biological vector control of mosquito-borne diseases. Lancet Infect Dis. 2011; 11: 84–85. PMID: 21351390
13. Slatko BE, Luck AN, Dobson SL, Foster JM. *Wolbachia* endosymbionts and human disease control. Mol Biochem Parasitol. 2014; 195: 88–95. doi: 10.1016/j.molbiopara.2014.07.004 PMID: 25046729
14. Bian G, Joshi D, Dong Y, Lu P, Zhou G, Pan X, et al. *Wolbachia* Invades *Anopheles stephensi* Populations and Induces Refractoriness to *Plasmodium* Infection. Science. 2013; 340: 748–751. doi: 10.1126/science.1236192 PMID: 23661760
15. Frentiu FD, Zakir T, Walker T, Popovici J, Pyke AT, van den Hurk A, et al. Limited dengue virus replication in field-collected *Aedes aegypti* mosquitoes infected with *Wolbachia*. PLoS Negl Trop Dis. 2014; 8: e2688. doi: 10.1371/journal.pntd.0002688 PMID: 24587459
16. Zug R, Hammerstein P. Still a host of hosts for *Wolbachia*: analysis of recent data suggests that 40% of terrestrial arthropod species are infected. PloS One. 2012; 7: e38544. doi: 10.1371/journal.pone.0038544 PMID: 22685581

17. Werren JH, Baldo L, Clark ME. *Wolbachia*: master manipulators of invertebrate biology. Nat Rev Microbiol. 2008; 6: 741–751. doi: 10.1038/nrmicro1969 PMID: 18794912

18. Bian G, Xu Y, Lu P, Xie Y, Xi Z. The endosymbiotic bacterium *Wolbachia* induces resistance to dengue virus in *Aedes aegypti*. PLoS Pathog. 2010; 6: e1000833. doi: 10.1371/journal.ppat.1000833 PMID: 20368968

19. Moreira LA, Iturbe-Ormaetxe I, Jeffery JA, Lu G, Pyke AT, Hedges LM, et al. A *Wolbachia* symbiont in *Aedes aegypti* limits infection with Dengue, Chikungunya, and *Plasmodium*. Cell. 2009; 139: 1268–1278. doi: 10.1016/j.cell.2009.11.042 PMID: 20064373

20. Van den Hurk AF, Hall-Mendelin S, Pyke AT, Frentiu FD, McElroy K, Day A, et al. Impact of *Wolbachia* on infection with chikungunya and yellow fever viruses in the mosquito vector *Aedes aegypti*. PLoS Negl Trop Dis. 2012; 6: e1892. doi: 10.1371/journal.pntd.0001892 PMID: 23133693

21. Hussain M, Lu G, Torres S, Edmonds JH, Kay BH, Khromykh AA, et al. Effect of *Wolbachia* on replication of West Nile virus in a mosquito cell line and adult mosquitoes. J Virol. 2013; 87: 851–858. doi: 10.1128/JVI.01837-12 PMID: 23115298

22. Zélé F, Nicot A, Berthomieu A, Weill M, Duron O, Rivero A. *Wolbachia* increases susceptibility to *Plasmodium* infection in a natural system. Proc Biol Sci. 2014; 281: 20132837. doi: 10.1098/rspb.2013.2837 PMID: 24500167

23. Zouache K, Voronin D, Tran-Van V, Mousson L, Failloux A-B, Mavingui P. Persistent *Wolbachia* and cultivable bacteria infection in the reproductive and somatic tissues of the mosquito vector *Aedes albopictus*. PloS One. 2009; 4: e6388. doi: 10.1371/journal.pone.0006388 PMID: 19633721

24. Zouache K, Raharimalala FN, Raquin V, Tran-Van V, Raveloson LHR, Ravelonandro P, et al. Bacterial diversity of field-caught mosquitoes, *Aedes albopictus* and *Aedes aegypti*, from different geographic regions of Madagascar. FEMS Microbiol Ecol. 2011; 75: 377–389. doi: 10.1111/j.1574-6941.2010.01012.x PMID: 21175696

25. Mousson L, Zouache K, Arias-Goeta C, Raquin V, Mavingui P, Failloux A-B. The Native *Wolbachia* Symbionts Limit Transmission of Dengue Virus in *Aedes albopictus*. PLoS Negl Trop Dis. 2012; 6: e1989. doi: 10.1371/journal.pntd.0001989 PMID: 23301109

26. Mousson L, Martin E, Zouache K, Madec Y, Mavingui P, Failloux AB. *Wolbachia* modulates Chikungunya replication in *Aedes albopictus*. Mol Ecol. 2010; 19: 1953–1964. doi: 10.1111/j.1365-294X.2010.04606.x PMID: 20345686

27. Caragata EP, Rancès E, Hedges LM, Gofton AW, Johnson KN, O'Neill SL, et al. Dietary cholesterol modulates pathogen blocking by *Wolbachia*. PLoS Pathog. 2013; 9: e1003459. doi: 10.1371/journal.ppat.1003459 PMID: 23825950

28. Caragata EP, Rancès E, O'Neill SL, McGraw EA. Competition for amino acids between *Wolbachia* and the mosquito host, *Aedes aegypti*. Microb Ecol. 2014; 67: 205–218. doi: 10.1007/s00248-013-0339-4 PMID: 24337107

29. Voronin D, Cook DAN, Steven A, Taylor MJ. Autophagy regulates *Wolbachia* populations across diverse symbiotic associations. Proc Natl Acad Sci U S A. 2012; 109: E1638–1646. doi: 10.1073/pnas.1203519109 PMID: 22645363

30. Brennan LJ, Keddie BA, Braig HR, Harris HL. The endosymbiont *Wolbachia* pipientis induces the expression of host antioxidant proteins in an *Aedes albopictus* cell line. PloS One. 2008; 3: e2083. doi: 10.1371/journal.pone.0002083 PMID: 18461124

31. Hussain M, Frentiu FD, Moreira LA, O'Neill SL, Asgari S. *Wolbachia* uses host microRNAs to manipulate host gene expression and facilitate colonization of the dengue vector *Aedes aegypti*. Proc Natl Acad Sci U S A. 2011; 108: 9250–9255. doi: 10.1073/pnas.1105469108 PMID: 21576469

32. Rancès E, Ye YH, Woolfit M, McGraw EA, O'Neill SL. The relative importance of innate immune priming in *Wolbachia*-mediated dengue interference. PLoS Pathog. 2012; 8: e1002548. doi: 10.1371/journal.ppat.1002548 PMID: 22383881

33. Hussain M, O'Neill SL, Asgari S. *Wolbachia* interferes with the intracellular distribution of Argonaute 1 in the dengue vector *Aedes aegypti* by manipulating the host microRNAs. RNA Biol. 2013; 10: 1868–1875. doi: 10.4161/rna.27392 PMID: 24351659

34. Hughes GL, Ren X, Ramirez JL, Sakamoto JM, Bailey JA, Jedlicka AE, et al. *Wolbachia* infections in *Anopheles gambiae* cells: transcriptomic characterization of a novel host-symbiont interaction. PLoS Pathog. 2011; 7: e1001296. doi: 10.1371/journal.ppat.1001296 PMID: 21379333

35. Frentiu FD, Robinson J, Young PR, McGraw EA, O'Neill SL. *Wolbachia*-mediated resistance to dengue virus infection and death at the cellular level. PloS One. 2010; 5: e13398. doi: 10.1371/journal.pone.0013398 PMID: 20976219

36. O'Neill SL, Pettigrew MM, Sinkins SP, Braig HR, Andreadis TG, Tesh RB. In vitro cultivation of *Wolbachia pipientis* in an *Aedes albopictus* cell line. Insect Mol Biol. 1997; 6: 33–39. PMID: 9013253

37. Schuffenecker I, Iteman I, Michault A, Murri S, Frangeul L, Vaney M-C, et al. Genome microevolution of chikungunya viruses causing the Indian Ocean outbreak. PLoS Med. 2006; 3: e263. doi: 10.1371/journal.pmed.0030263 PMID: 16700631

38. Dubrulle M, Mousson L, Moutailler S, Vazeille M, Failloux A-B. Chikungunya virus and *Aedes* mosquitoes: saliva is infectious as soon as two days after oral infection. PloS One. 2009; 4: e5895. doi: 10.1371/journal.pone.0005895 PMID: 19521520

39. Payne AF, Binduga-Gajewska I, Kauffman EB, Kramer LD. Quantitation of flaviviruses by fluorescent focus assay. J Virol Methods. 2006; 134: 183–189. doi: 10.1016/j.jviromet.2006.01.003 PMID: 16510196

40. Venard CM-P, Crain PR, Dobson SL. SYTO11 staining vs FISH staining: a comparison of two methods to stain *Wolbachia pipientis* in cell cultures. Lett Appl Microbiol. 2011; 52: 168–176. doi: 10.1111/j.1472-765X.2010.02986.x PMID: 21214605

41. Fenollar F, La Scola B, Inokuma H, Dumler JS, Taylor MJ, Raoult D. Culture and phenotypic characterization of a *Wolbachia pipientis* isolate. J Clin Microbiol. 2003; 41: 5434–5441. PMID: 14662922

42. Voronin D, Tran-Van V, Potier P, Mavingui P. Transinfection and growth discrepancy of *Drosophila Wolbachia* strain *w*Mel in cell lines of the mosquito *Aedes albopictus*. J Appl Microbiol. 2010; 108: 2133–2141. doi: 10.1111/j.1365-2672.2009.04621.x PMID: 19951376

43. Osborne SE, Leong YS, O'Neill SL, Johnson KN. Variation in antiviral protection mediated by different *Wolbachia* strains in *Drosophila simulans*. PLoS Pathog. 2009; 5: e1000656. doi: 10.1371/journal.ppat.1000656 PMID: 19911047

44. Raquin V, Wannagat M, Zouache K, Legras-Lachuer C, Moro CV, Mavingui P. Detection of dengue group viruses by fluorescence *in situ* hybridization. Parasit Vectors. 2012; 5: 243. doi: 10.1186/1756-3305-5-243 PMID: 23110979

45. Bonilauri P, Bellini R, Calzolari M, Angelini R, Venturi L, Fallacara F, et al. Chikungunya virus in *Aedes albopictus*, Italy. Emerg Infect Dis. 2008; 14: 852–854. doi: 10.3201/eid1405.071144 PMID: 18439383

46. Tsetsarkin KA, Chen R, Yun R, Rossi SL, Plante KS, Guerbois M, et al. Multi-peaked adaptive landscape for chikungunya virus evolution predicts continued fitness optimization in *Aedes albopictus* mosquitoes. Nat Commun. 2014; 5: 4084. doi: 10.1038/ncomms5084 PMID: 24933611

47. Delatte H, Paupy C, Dehecq JS, Thiria J, Failloux AB, Fontenille D. [*Aedes albopictus*, vector of chikungunya and dengue viruses in Reunion Island: biology and control]. Parasite Paris Fr. 2008; 15: 3–13.

48. Ponlawat A, Scott JG, Harrington LC. Insecticide susceptibility of *Aedes aegypti* and *Aedes albopictus* across Thailand. J Med Entomol. 2005; 42: 821–825. PMID: 16363166

49. Blagrove MSC, Arias-Goeta C, Di Genua C, Failloux A-B, Sinkins SP. A *Wolbachia w*Mel transinfection in *Aedes albopictus* is not detrimental to host fitness and inhibits Chikungunya virus. PLoS Negl Trop Dis. 2013; 7: e2152. doi: 10.1371/journal.pntd.0002152 PMID: 23556030

50. Blagrove MSC, Arias-Goeta C, Failloux A-B, Sinkins SP. *Wolbachia* strain *w*Mel induces cytoplasmic incompatibility and blocks dengue transmission in *Aedes albopictus*. Proc Natl Acad Sci U S A. 2012; 109: 255–260. doi: 10.1073/pnas.1112021108 PMID: 22123944

51. Lu P, Bian G, Pan X, Xi Z. *Wolbachia* Induces Density-Dependent Inhibition to Dengue Virus in Mosquito Cells. PLoS Negl Trop Dis. 2012; 6: e1754. doi: 10.1371/journal.pntd.0001754 PMID: 22848774

52. McMeniman CJ, Lane AM, Fong AWC, Voronin DA, Iturbe-Ormaetxe I, Yamada R, et al. Host adaptation of a *Wolbachia* strain after long-term serial passage in mosquito cell lines. Appl Environ Microbiol. 2008; 74: 6963–6969. doi: 10.1128/AEM.01038-08 PMID: 18836024

53. Fallon AM, Witthuhn BA. Proteasome activity in a naïve mosquito cell line infected with *Wolbachia pipientis w*AlbB. In Vitro Cell Dev Biol Anim. 2009; 45: 460–466. doi: 10.1007/s11626-009-9193-6 PMID: 19296184

54. Fallon AM, Baldridge GD, Higgins LA, Witthuhn BA. *Wolbachia* from the planthopper *Laodelphax striatellus* establishes a robust, persistent, streptomycin-resistant infection in clonal mosquito cells. In Vitro Cell Dev Biol Anim. 2013; 49: 66–73. doi: 10.1007/s11626-012-9571-3 PMID: 23271364

55. Khoo CCH, Venard CMP, Fu Y, Mercer DR, Dobson SL. Infection, growth and maintenance of *Wolbachia pipientis* in clonal and non-clonal *Aedes albopictus* cell cultures. Bull Entomol Res. 2013; 103: 251–260. doi: 10.1017/S0007485312000648 PMID: 23113940

56. Noda H, Miyoshi T, Koizumi Y. In vitro cultivation of *Wolbachia* in insect and mammalian cell lines. In Vitro Cell Dev Biol Anim. 2002; 38: 423–427. doi: 10.1290/1071-2690(2002)038<0423:IVCOWI>2.0.CO;2 PMID: 12534342

57. Salazar MI, Richardson JH, Sánchez-Vargas I, Olson KE, Beaty BJ. Dengue virus type 2: replication and tropisms in orally infected *Aedes aegypti* mosquitoes. BMC Microbiol. 2007; 7: 9. doi: 10.1186/1471-2180-7-9 PMID: 17263893

58. Osborne SE, Iturbe-Ormaetxe I, Brownlie JC, O'Neill SL, Johnson KN. Antiviral Protection and the Importance of *Wolbachia* Density and Tissue Tropism in *Drosophila simulans*. Appl Environ Microbiol. 2012; 78: 6922–6929. doi: 10.1128/AEM.01727-12 PMID: 22843518

59. Kingsolver MB, Huang Z, Hardy RW. Insect antiviral innate immunity: pathways, effectors, and connections. J Mol Biol. 2013; 425: 4921–4936. doi: 10.1016/j.jmb.2013.10.006 PMID: 24120681

60. Brackney DE, Scott JC, Sagawa F, Woodward JE, Miller NA, Schilkey FD, et al. C6/36 *Aedes albopictus* cells have a dysfunctional antiviral RNA interference response. PLoS Negl Trop Dis. 2010; 4: e856. doi: 10.1371/journal.pntd.0000856 PMID: 21049065

61. Zhang G, Hussain M, O'Neill SL, Asgari S. *Wolbachia* uses a host microRNA to regulate transcripts of a methyltransferase, contributing to dengue virus inhibition in *Aedes aegypti*. Proc Natl Acad Sci U S A. 2013; 110: 10276–10281. doi: 10.1073/pnas.1303603110 PMID: 23733960

62. Zhang G, Hussain M, Asgari S. Regulation of arginine methyltransferase 3 by a *Wolbachia*-induced microRNA in *Aedes aegypti* and its effect on *Wolbachia* and dengue virus replication. Insect Biochem Mol Biol. 2014; 53: 81–88. doi: 10.1016/j.ibmb.2014.08.003 PMID: 25158106

63. Kremer N, Voronin D, Charif D, Mavingui P, Mollereau B, Vavre F. *Wolbachia* interferes with ferritin expression and iron metabolism in insects. PLoS Pathog. 2009; 5: e1000630. doi: 10.1371/journal.ppat.1000630 PMID: 19851452

64. Schaible UE, Kaufmann SHE. Iron and microbial infection. Nat Rev Microbiol. 2004; 2: 946–953. doi: 10.1038/nrmicro1046 PMID: 15550940

65. Krejbich-Trotot P, Gay B, Li-Pat-Yuen G, Hoarau J-J, Jaffar-Bandjee M-C, Briant L, et al. Chikungunya triggers an autophagic process which promotes viral replication. Virol J. 2011; 8: 432. doi: 10.1186/1743-422X-8-432 PMID: 21902836

66. Baldridge GD, Baldridge AS, Witthuhn BA, Higgins L, Markowski TW, Fallon AM. Proteomic profiling of a robust *Wolbachia* infection in an *Aedes albopictus* mosquito cell line. Mol Microbiol. 2014; 94: 537–556. doi: 10.1111/mmi.12768 PMID: 25155417

67. Darby AC, Christina Gill A, Armstrong SD, Hartley CS, Xia D, Wastling JM, et al. Integrated transcriptomic and proteomic analysis of the global response of *Wolbachia* to doxycycline-induced stress. ISME J. 2014; 8: 925–937. doi: 10.1038/ismej.2013.192 PMID: 24152719

68. Kambris Z, Blagborough AM, Pinto SB, Blagrove MSC, Godfray HCJ, Sinden RE, et al. *Wolbachia* stimulates immune gene expression and inhibits *Plasmodium* development in *Anopheles gambiae*. PLoS Pathog. 2010; 6: e1001143. doi: 10.1371/journal.ppat.1001143 PMID: 20949079

69. Ramirez JL, Short SM, Bahia AC, Saraiva RG, Dong Y, Kang S, et al. *Chromobacterium Csp*_P Reduces Malaria and Dengue Infection in Vector Mosquitoes and Has Entomopathogenic and In Vitro Anti-pathogen Activities. PLoS Pathog. 2014; 10: e1004398. doi: 10.1371/journal.ppat.1004398 PMID: 25340821

70. Bourtzis K, Dobson SL, Xi Z, Rasgon JL, Calvitti M, Moreira LA, et al. Harnessing mosquito-*Wolbachia* symbiosis for vector and disease control. Acta Trop. 2014; 132 Suppl: S150–163. doi: 10.1016/j.actatropica.2013.11.004 PMID: 24252486

71. Fansiri T, Fontaine A, Diancourt L, Caro V, Thaisomboonsuk B, Richardson JH, et al. Genetic mapping of specific interactions between *Aedes aegypti* mosquitoes and dengue viruses. PLoS Genet. 2013; 9: e1003621. doi: 10.1371/journal.pgen.1003621 PMID: 23935524

72. Richards SL, Anderson SL, Lord CC, Tabachnick WJ. Effects of virus dose and extrinsic incubation temperature on vector competence of *Culex nigripalpus* (*Diptera*: *Culicidae*) for St. Louis encephalitis virus. J Med Entomol. 2012; 49: 1502–1506. PMID: 23270182

73. Carrington LB, Seifert SN, Armijos MV, Lambrechts L, Scott TW. Reduction of *Aedes aegypti* vector competence for dengue virus under large temperature fluctuations. Am J Trop Med Hyg. 2013; 88: 689–697. doi: 10.4269/ajtmh.12-0488 PMID: 23438766

74. Jupatanakul N, Sim S, Dimopoulos G. The Insect Microbiome Modulates Vector Competence for Arboviruses. Viruses. 2014; 6: 4294–4313. doi: 10.3390/v6114294 PMID: 25393895

75. Murdock CC, Blanford S, Hughes GL, Rasgon JL, Thomas MB. Temperature alters Plasmodium blocking by *Wolbachia*. Sci Rep. 2014; 4: 3932. doi: 10.1038/srep03932 PMID: 24488176

76. Hughes GL, Dodson BL, Johnson RM, Murdock CC, Tsujimoto H, Suzuki Y, et al. Native microbiome impedes vertical transmission of *Wolbachia* in *Anopheles mosquitoes*. Proc Natl Acad Sci U S A. 2014; 111: 12498–12503. doi: 10.1073/pnas.1408888111 PMID: 25114252

77. Tortosa P, Courtiol A, Moutailler S, Failloux A-B, Weill M. Chikungunya-*Wolbachia* interplay in *Aedes albopictus*. Insect Mol Biol. 2008; 17: 677–684. doi: 10.1111/j.1365-2583.2008.00842.x PMID: 19133077

78. Vazeille M, Moutailler S, Coudrier D, Rousseaux C, Khun H, Huerre M, et al. Two Chikungunya isolates from the outbreak of La Reunion (Indian Ocean) exhibit different patterns of infection in the mosquito, *Aedes albopictus*. PloS One. 2007; 2: e1168. doi: 10.1371/journal.pone.0001168 PMID: 18000540

79. Sanguin H, Herrera A, Oger-Desfeux C, Dechesne A, Simonet P, Navarro E, et al. Development and validation of a prototype 16S rRNA-based taxonomic microarray for *Alphaproteobacteria*. Environ Microbiol. 2006; 8: 289–307. doi: 10.1111/j.1462-2920.2005.00895.x PMID: 16423016

80. Heddi A, Grenier AM, Khatchadourian C, Charles H, Nardon P. Four intracellular genomes direct weevil biology: nuclear, mitochondrial, principal endosymbiont, and *Wolbachia*. Proc Natl Acad Sci U S A. 1999; 96: 6814–6819. PMID: 10359795
